# Selection of optimal extraction and RT-PCR protocols for stool RNA detection of colorectal cancer associated immune genes

**DOI:** 10.1038/s41598-024-78680-0

**Published:** 2024-11-10

**Authors:** Thura Akrem Omran, Inger Line Madsø, Per Christian Sæther, Vahid Bemanian, Hege Smith Tunsjø

**Affiliations:** 1 Department of Life Sciences and Health, Oslo Metropolitan University, Oslo, Norway; 2 Department of Pathology, Akershus University Hospital, Lørenskog, Norway; 3 Department of Immunology and Transfusion Medicine, Akershus University Hospital, Lørenskog, Norway

**Keywords:** Colorectal cancer, Non-invasive detection, Stool mRNA biomarkers, Diagnostic method, Cancer, Immunology, Biomarkers, Colorectal cancer

## Abstract

There is a growing interest in using fecal mRNA transcripts as biomarkers for non-invasive detection of colorectal cancer (CRC). The following study compares different RNA extraction and reverse transcription PCR (RT-PCR) methods for mRNA detection in stool and identifies a robust and sensitive protocol. A combination of the Stool total RNA purification kit (Norgen) and the Superscript III one-step RT-PCR kit (Invitrogen) provided high RNA purity and sensitive and consistent mRNA detection, making them well-suited candidates for large-scale studies. We tested the protocol by detecting the mRNA of several immune genes (*CXCL1*, *IL8*, *IL1B*, *IL6*, *PTGS2*, and *SPP1*) in 22 CRCs, 24 adenomatous polyps, and 22 control stool samples. All these inflammatory markers, except for *CXCL1*, showed a strong association with CRC. Cancer stool samples showed increased levels of *IL1B*, *IL8*, and *PTGS2* transcripts compared to polyp and control groups. Thus, this work supports the potential use of fecal mRNA as biomarkers for CRC detection.

**Supplementary Information:**

The online version contains supplementary material available at 10.1038/s41598-024-78680-0.

## Introduction

Colorectal cancer (CRC) ranks as one of the most common malignancies globally^1^. It evolves gradually from normal intestinal mucosa into benign precancerous adenomas, then to carcinoma, and ultimately to aggressive metastatic disease over several years^2,3^. When tumors are detected early and remain localized, the 5-year relative survival rate is approximately 90%. However, in the case of late-stage detection, the survival chances for patients drastically decrease to approximately 10% due to metastasis^4,5^. Given the fact that early detection results in large positive changes in morbidity and mortality rates, several- countries have programs for early detection. Norway implemented a screening program in 2022^6^. Although colonoscopy remains the gold standard for diagnosis, it is both invasive and costly. Consequently, the fecal immunochemical test (FIT) is often used for its non-invasiveness. While it has been shown to reduce mortality worldwide, FIT sensitivity is variable, especially for tumors in the ascending colon and advanced adenomas^7–9^.

To improve the accuracy of stool-based CRC screening, more research is being done on different biomolecules, such as microbial markers, proteins, DNA, and RNA^10–12^. Due to the important role of the immune system in CRC, immunological genes activated during CRC could be promising candidates for screening^13^. Previous studies, including those conducted by our group, have indicated an upregulation of the genes like Dual Specificity Phosphatase 4 (*DUSP4*), S100 Calcium Binding Protein A8 (*S100A8*), S100 Calcium Binding Protein A9 (*S100A9*), C-X-C Motif Chemokine Ligand 1 (*CXCL1*), C-X-C Motif Chemokine Ligand 8 (*CXCL8* or *IL8*), Interleukin 1 Beta (*IL1B*), Prostaglandin-Endoperoxide Synthase 2 (*PTGS2* or *COX2*), Secreted Phosphoprotein 1 (*SPP1*), and Interleukin 6 (*IL6*) in tumor tissues compared to healthy tissue from the same patients^14–17^. Our earlier research also indicated increased levels of certain genes at the polyp stage, suggesting their potential utility in early detection^17^. Some of these genes are known markers in inflammatory diseases like inflammatory bowel disease (IBD), while others may be more specifically associated with cancer progression^18,19^.

Stool samples, being in direct contact with the colonic epithelium, arguably represent one of the best materials to screen, since they may well be the closest representation of the colonic cellular environment. The colonic epithelium represents one of the most dynamic cellular systems in the human body and is reported to be renewed every 3–5 days throughout life to ensure barrier integrity^20^. This renewal is tightly regulated by a complex network, which includes various proteins, signaling molecules, and structural components situated within the subepithelial extracellular matrix, maintains a dynamic balance between cell division, differentiation, migration, and cell death^20,21^. Cell death typically occurs through apoptosis, after which these apoptotic cells are resorbed by neighboring colonocytes and macrophages. In neoplastic progression, alterations occur in the regulation of apoptosis and cell-cell adhesion within the neoplastic tissues. This results in increased shedding of epithelial cells compared to healthy mucosa^22,23^.

Compared to other biomolecules, there have been few studies on mRNA detection in stool samples, possibly due to mRNA’s instability and the challenging stool environment, which is rich in microbes and bacterial transcripts^24,25^. During the last few years, however, several studies have shown that stool-derived eukaryotic RNA is a promising biomarker for the detection of CRC^26–31^. In 2020, Beaulieu and Herring proposed that mRNA is preserved in cancer cells shed from the colorectum, contrasting with its degradation in apoptotic enterocytes^32^. This potentially facilitates the advantageous detection of cancer-derived mRNA in stool samples. Exploiting their further potential as biomarkers requires reliable and robust methods for their detection, including user-friendly RNA extraction methods^33^. Considerable variation exists between studies for RNA extraction protocols: while some have used phenol/guanidine-based manual extraction protocols^27,32^, others have utilized automated extraction systems based on silica-coated paramagnetic beads^29^. Also, in some studies, colonocyte enrichment or differential centrifugation prior to the RNA extraction was performed^34,35^. In addition, the low abundance of human mRNA in stool, relative to that of microbial RNA, makes it necessary to use very sensitive approaches for their detection. A variety of reverse transcription PCR (RT-PCR) strategies have been pursued, which include pre-amplification and increasing the number of PCR cycles^27,31^. However, these RT-PCR methods must maintain high specificity to avoid cross-reactivity with microbial DNA and RNA.

Despite the increasing recognition of mRNA transcripts as promising biomarkers for CRC, methodological difficulties in detecting these transcripts in stool samples remain. In order for CRC- specific mRNA to be employed effectively as biomarkers for CRC detection, there is a need for the development of sensitive, reliable, and user-friendly protocols for both extraction and the detection of the mRNA from stool samples. The primary objective of the present study was to evaluate the impact of different extraction methods and RT-PCR protocols for the detection of mRNA in stool samples. The secondary objective was to test the optimal protocol for several CRC-associated immune genes and to compare these between colorectal cancer patients, patients with adenomatous polyps, and healthy controls.

## Methods

### Patients and stool samples

**Fig. 1:**
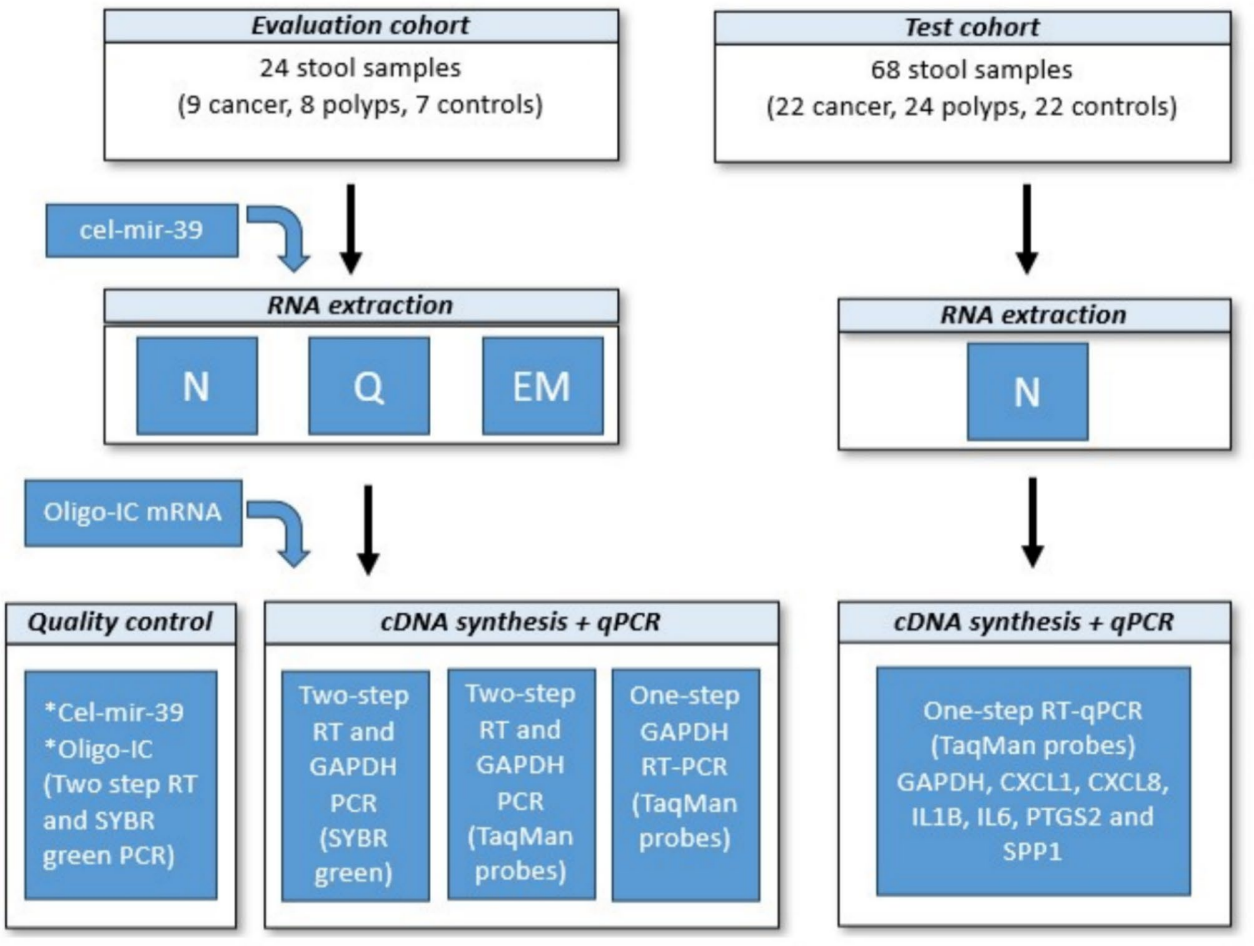
Study design for RNA extraction and PCR methods. The figure outlines the process used in the study of RNA extraction and PCR methods from stool samples in two cohorts: an evaluation cohort (24 samples: 9 cancer, 8 polyps, 7 controls) and a test cohort (68 samples: 22 cancer, 24 polyps, 22 controls). The evaluation cohort involved multiple RNA extraction methods: Norgen (N), Qiagen (Q), and EasyMAG BioMérieux (EM), with subsequent quality control steps using cel-mir-39 and Oligo-IC mRNA. The PCR methods included two-step RT and *GAPDH* PCR with a DNA intercalating dye (SYBR green) and TaqMan probes, and one-step *GAPDH* RT-PCR with TaqMan probes. In the test cohort, RNA extraction was performed using Norgen (N), followed by one-step RT-PCR with TaqMan probes targeting *GAPDH, CXCL1, IL18, IL1B, IL6, PTGS2*, and *SPP1*.

Methodological comparisons were carried out using an evaluation cohort with stool samples from 24 patients (nine cancers, eight polyps, and seven controls) who underwent colonoscopy procedures at Akershus University Hospital (Ahus) from 2022 to 2023. The evaluation cohort included samples from various groups, ensuring a thorough and representative technical evaluation, eliminating the need for specific patient data at this stage. After establishing the best performing protocol for RNA extraction and RT-PCR analysis, the chosen protocol was tested with 68 stool samples from a test cohort of patients (22 cancer, 24 polyps, and 22 controls) who underwent colonoscopy from 2014 to 2017 (Table 1). The study design is depicted in Fig. 1. Stool samples were collected prior to bowel preparation or 1–2 weeks after colonoscopy and were immediately preserved in RNAlater and stored at -80 °C after 1–3 days. All samples for both the evaluation and test cohorts were defrosted and extracted during the course of this study. Colonoscopies were scheduled for various medical reasons, such as gastrointestinal bleeding, weight loss, alterations in bowel habits, or the detection of polyps or malignancies through CT colonography. Following findings from the colonoscopy, the patients were grouped into cancer, adenomatous polyps ≥ 10 mm, or controls. Participants with a history of inflammatory bowel disease were excluded from the study. All samples were collected during the initial colonoscopy, prior to the diagnosis. Prior to colonoscopy, the patients were invited to participate in the study and received written and oral information about the additional samples that would be collected and about their rights to withdraw from the study at any time. The study was approved by the data protection manager at Akershus University Hospital (Ahus) and the Regional Committee for Medical and Health Research Ethics, Southeast Norway (Approval Number: 2012/1944). The research was performed in accordance with the relevant guidelines and regulations, including the declaration of Helsinki.

**Table 1:** Clinical and demographic data of study participants in the test cohort.

Characteristics	Cancer (*n* = 22)	Polyp (*n* = 24)	Control (*n* = 22)
Age in years (average)	69.0	66.9	58.5
Sex (F/M)	6/16	13/11	9/13
Weight in kg (missing)	77.9 (3)	82.9 (4)	80.9 (2)
Smoking (yes/no/missing)	(2/17/3)	(2/20/2)	(2/18/2)
Vomiting or diarrhea (yes/no/missing)	(6/13/3)	(3/19/2)	(11/9/2)
Antibiotics (last 3 mo.) (yes/no/missing)	(2/17/3)	(4/19/2)	(1/19/2)
Location of tumor: n (%)			
Cecum	6 (27%)	7 (29%)	-
Ascending colon	2 (9%)	1 (4%)	-
Transverse colon	2 (9%)	5 (21%)	-
Sigmoid colon	7 (32%)	9 (38%)	-
Rectosigmoid junction	2 (9%)	1(4%)	-
Rectum	3 (14%)	1(4%)	-
Histologic diagnosis			
Adenocarcinoma	22	-	-
Tubular, high-grade dysplasia	-	1	-
Tubular, low-grade dysplasia	-	18	-
Tubulovillous, low-grade dysplasia	-	3	-
Unknown*	-	2	-

*The sample used for histological classification of the tissue had few cells and was insufficient for histological diagnosis.

### RNA extraction from stool samples using three different methods

The 24 stool samples from the evaluation cohort were used to extract RNA with three different extraction methods: Stool total RNA purification kit (Norgen Biotech Corp., Ontario, Canada), miRNeasy Mini kit (Qiagen, Hilden, Germany), and the NucliSENS EasyMAG system with the generic protocol for stool samples (BioMérieux, Lyon, France). The methods were chosen based on different extraction principles, as described in Table 2.

Standardization of the extraction process was achieved by using 200 µL of stool preserved in RNAlater combined with the respective lysis buffer from each kit (Table 2). The mixtures were homogenized in respective bead tubes using a Vortex Genie 2 (Scientific Industries, Bohemia, NY) at a speed of 2,850 rpm for five minutes (Table 2).

Following homogenization, the protocols showed minor variations. In the Norgen and EasyMAG protocols, the tubes underwent centrifugation at 17,000 g for a duration of three minutes. Afterward, 600 µL of the supernatant was carefully transferred to a new tube for extraction, following the manufacturer’s specifications. In the Qiagen protocol, tubes were kept at room temperature for 5 min, then added 140 µl of chloroform and mixed thoroughly for phase separation. Tubes were centrifuged at 12,000 g at 4 °C for 15 min before the supernatant (350 µL) was transferred for further extraction following instructions from the manufacturer. For quality control of the extraction processes, 3 µl of cel-miR-39 from the microRNA Cel-miR-39 Spike-in kit (Norgen Biotek) was added to the supernatant of each sample prior to further processing. RNA extraction was performed in duplicate for each sample in all three extraction kits.

### DNase treatment to eliminate DNA contamination

The removal of DNA contamination from feces samples was achieved through specific enzymatic procedures, ensuring the purity of the RNA. The Norgen and Qiagen kits utilized the Qiagen RNase-Free DNase Set for the samples. This treatment involved applying the kit components directly to the spin columns to degrade any potential DNA contaminants. For the nucleic acids obtained through the EasyMAG (EM) system, RQ1 RNase-Free DNase (Promega, Madison, WI, USA) was used for DNA degradation following the instructions from the manufacturer.

**Table 2:** Overview of RNA extraction kits and protocols.

Extraction kit	Bead tubes and lysis	RNA extraction principle	Molecules extracted	Time used for 24 samples
Stool total RNA purification (Norgen)	Bead tube (Norgen Biotek) with silica beads and 1 mL of non-ionic detergent-based lysis buffer C	Spin-column-based solid-phase extraction (resin separation matrix)	miRNA, mRNA rRNA	Approx. 180 min
miRNeasy mini kit (Qiagen)	Lysing matrix E tubes containing ceramic, silica, and metal beads. (MP Biomedicals (MPbio), CA, USA)), 700 µl QIAzol Lysis Reagent	Phase separation with organic solvents (chloroform, phenol) followed by spin-column-based solid-phase extraction (silica gel)	miRNA, mRNA rRNA	Approx. 180 min
NucliSENS EasyMAG (BioMérieux)	Lysing matrix E tubes with ceramic, silica and metal beads (MPbio) combined with 1 ml of NucliSENS lysis buffer (guanidinium thiocyanate-based)	Magnetic bead-based solid-phase extraction (silica-coated paramagnetic beads)	mRNA, rRNA, DNA*	Approx. 60 min

*DNA must be removed after nucleic acid extraction.

### Assessment of RNA quantities and qualities

To measure the concentration and evaluate the purity of RNA extracted from stool samples, the Nanodrop 2000 spectrophotometer (Thermo Fisher Scientific, Waltham, MA, USA) and the Qubit 3.0 fluorometer with a Qubit RNA HS assay kit (Life Technologies, CA, USA) were employed. Furthermore, for a selection of samples from all kits, the integrity and size distribution of the RNA molecules were analyzed using the Agilent 2100 Bioanalyzer (Agilent, Palo Alto, CA, USA), along with the Agilent RNA 6000 Nano Kit.

### Real-time reverse transcription PCR protocols

Based on previous results with high expression of the housekeeping gene glyceraldehyde phosphate dehydrogenase (*GAPDH*) in mucosal samples, this target was selected for analysis in stool^17^. Three RT-PCR protocols were evaluated. First protocol was a two-step procedure based on cDNA synthesis with the iScript cDNA synthesis kit (Bio-Rad Laboratories, CA, USA), followed by PCR with SYBR green detection using the QuantiNova SYBR Green PCR kit (Qiagen, Hilden, Germany) and primers from Origene qstar (Table 3). The second protocol was also a two-step protocol with the same iScript cDNA synthesis, followed by a TaqMan probe-PCR using Brilliant III Ultra-Fast QPCR Master Mix (Agilent) and a premade *GAPDH* assay from ThermoFisher (Table 3). The third protocol was a one-step protocol using the Superscript III one-step RT-PCR kit (Invitrogen) and the TaqMan probe *GAPDH* assay from Thermofisher. Details of each protocol are described in Table 3. Quality controls: Negative controls were included for all assays and with each set-up. To test for false-positive results due to genomic DNA contamination, samples were also tested without reverse transcription. Additionally, agarose gel electrophoresis was performed to check for extra bands, and melting curve analysis was performed for the SYBR green assay. To assess for successful cDNA synthesis, RNA from all samples was spiked with 1 µl of the 200 base-pair RNA control (oligo-IC) (Qiagen) prior to reverse transcription, and the QuantiNova SYBR Green PCR kit (Qiagen) was used to detect this control oligo. To detect the cel-miR-39 “spike-in” control RNA that was added prior to RNA extraction, primers from the same kit (microRNA Cel-miR39 Spike-In Kit, Norgen Biotek) were applied with the miRCURY LNA RT kit and miRCURY LNA miRNA SYBR Green PCR kit for detection (Qiagen).

**Table 3:** Overview of RT-PCR kits and protocols.

cDNA synthesis	PCR	One-step/two-step	Primer/probe assays detection chemistry
250 ng RNA*. iScript cDNA synthesis kit (Biorad). 20 µl volume. Random hexamer primers.	2 µl undiluted cDNA (approx. 25 ng) Quantinova SYBR Green PCR Kit (Qiagen)	Two-step RT-PCR. RT: 25° C 5 min, 46 °C, 20 min, 95 °C 1 min. PCR: 40 cycles of 5 Sect. 95 °C, 10 Sect. 60 °C.	SYBR green *GAPDH*: Origene Qstar NM002046 F: 5’ GTCTCCTCTGACTTCAACAGCG-3 R: 5’ACCACCCTGTTGCTGTAGCCAA-3’ (spans exons 7–8)
250 ng RNA*. IScript cDNA synthesis kit (Biorad). 20 µl volume. Random hexamer primers.	2 µl undiluted cDNA (approx. 25 ng) Brilliant III Ultra-Fast QPCR Master Mix (Agilent).	Two-step RT-PCR. RT: 25 °C 5 min, 46 °C, 20 min, 95 C° 1 min. PCR: 40 cycles of 15 Sect. 95 °C, 30 Sect. 60 °C.	TaqMan hydrolysis probes *GAPDH*: Hs99999905_m1 (spans exon 2–3, amplicon length 122 bp)
25 ng (2 µl) RNA. Superscript III one-step RT-PCR (Invitrogen). Targeted primers only.		One-step RT-PCR: 50 °C, 20 min, 40 cycles of 15 Sect. 94 °C, 30 Sect. 60 °C	TaqMan hydrolysis probes *GAPDH*: Hs99999905_m1 (spans exon 2–3, amplicon length 122 bp)

*The RT kit has a maximum capacity of 1 µg of RNA. Different concentrations of RNA (125 ng–1 µg) and dilutions of cDNA (1:10 and undiluted) were tested to obtain the lowest Ct values without RT- or PCR-inhibition.

### Detection of cancer-associated gene transcripts in stool from cancer, polyp, and control groups

Based on the results from the methodological comparisons, RNA extraction with Norgen and one-step Superscript III RT-PCR were used for the analysis of the test cohort with 68 patient samples from three groups: cancer patients (*n* = 22), polyp patients (*n* = 24), and controls (*n* = 22) (Table 1). The immune genes selected for this study were *CXCL1*, *IL1B*, *IL6*, *IL8* (*CXCL8*), *PTGS2*, and *SPP1*. These genes had shown significantly higher expression in CRC tumor tissues according to our previous studies^17^. The following primer/probe assays were used: *CXCL1* Assay ID: Hs00236937_m1, IL1B Assay ID: Hs01555410_m1, *IL6* Assay ID: Hs00174131_m1, *IL8*/*CXCL8* Assay ID: Hs00174103_m1, *PTGS2* Assay ID: Hs00153133_m1, and *SPP1* Assay ID: Hs00959010_m1 (ThermoFisher). All RT-PCR experiments were performed in technical duplicates. PCR efficiency was evaluated and corrected for each PCR assay using LinRegPCR^36^. Negative controls were included in all experiments.

### Statistical analysis

To assess the normality of the distribution of differences between the extraction methods, the Shapiro-Wilk test was employed. The criteria for a normal distribution were not met across all analyses; hence, subsequent analyses utilized a non-parametric statistical approach. For comparative evaluation of the extraction methods and different PCR protocols, box plots were constructed, and a one-way repeated measures analysis of variance by ranks, specifically Friedman’s test for non-parametric samples, was performed. To determine which specific methods were significantly different, we performed post-hoc pairwise comparisons using Dunn’s test.

In the test cohort, variations in sample quantity were normalized using *GAPDH*. Transcription profiles were compared using the 2^−ΔCt^ method^37^, with normality tested by Shapiro-Wilk. Statistical differences across groups were assessed using the Kruskal-Wallis test and specific comparisons through the Mann-Whitney U test.

Statistical analyses were conducted using Python (version 3.10.9). The following libraries were utilized: pandas for data manipulation and analysis^38^, numpy for numerical operations^39^, matplotlib for data visualization^40^, scikit-learn for machine learning and statistical modeling^41^, and scipy for scientific and technical computing^42^.

## Results

### Comparison of stool RNA extraction methods

**Fig. 2:**
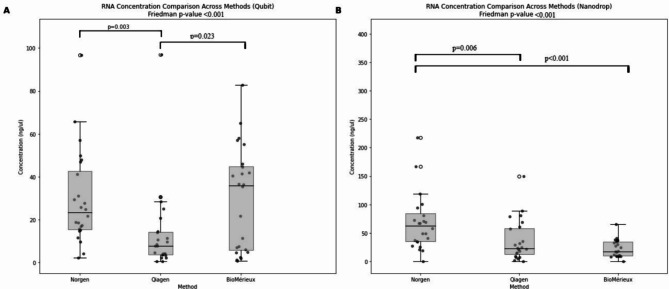
RNA concentration comparison across extraction methods. (**A**) Qubit measurements showed significant differences in RNA concentrations between the methods. Norgen yielded significantly higher RNA concentrations compared to Qiagen (*p* = 0.003). RNA concentrations differed significantly also between BioMérieux and Qiagen (*p* = 0.023). (**B**) Nanodrop measurement shows that the Norgen method yielded higher RNA concentrations compared to Qiagen (*p* = 0.006) and BioMérieux (*p* < 0.001). The Friedman p-values for both methods indicated overall significant differences across the extraction methods (Qubit p-value < 0.001; Nanodrop p-value < 0.001).

**Fig. 3:**
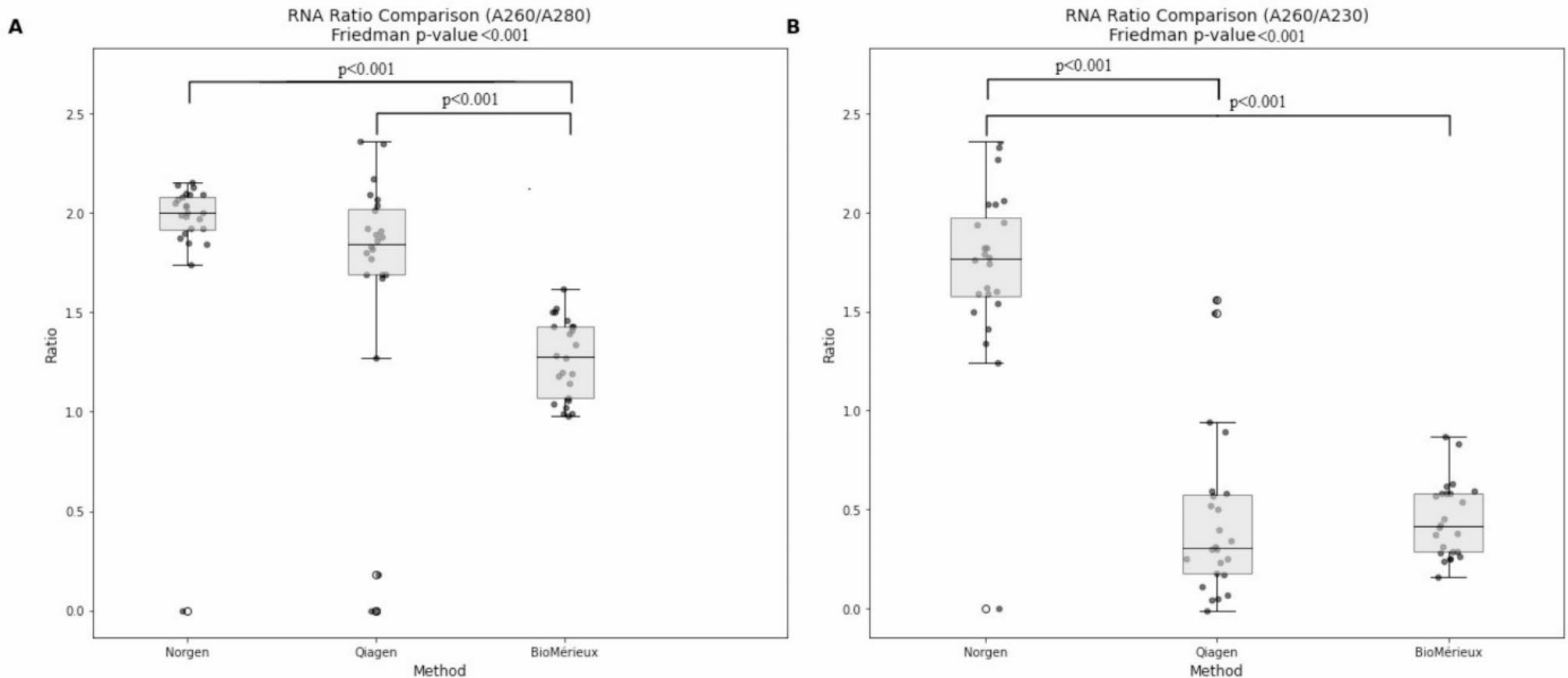
RNA purity ratio comparison across extraction methods. (**A**) RNA purity ratio (A260/A280). The Norgen extraction method exhibited the highest RNA purity, significantly surpassing both Qiagen and BioMérieux (p value < 0.001 for both comparisons). (**B**) RNA purity ratio (A260/A230). Similarly, the Norgen method achieved the highest RNA purity compared to Qiagen and BioMérieux, with significant differences (p value < 0.001 for both comparisons). The Friedman p-value < 0.001 confirms the significant variation in RNA purity across the extraction methods.

In a comparative analysis of RNA extraction kits, the study identified notable differences in performance among the kits tested. The Qubit method showed that measured RNA concentrations from the Norgen and BioMérieux extraction methods were significantly higher than those from Qiagen (p values 0.003 and 0.023, respectively) (Fig. 2A; Table 4). The Nanodrop method showed that the Stool Total RNA Purification kit from Norgen yielded higher RNA concentrations in comparison to the Qiagen and BioMérieux methods, with statistically significant differences (p value 0.006 and p value < 0.001, respectively) (Fig. 2B; Table 4). Moreover, with mean absorption ratios of 1.91 (260/280) and 1.71 (260/230), the Norgen kit showed the highest purity of the preparations. The A260/A230 absorption ratios for the Qiagen and BioMérieux (EM) kits showed no statistically significant difference in RNA purity (Fig. 3; Table 4). To evaluate the RNA integrity and size distribution of ribosomal RNA (rRNA) molecules from each kit, a selection of samples was analyzed with Agilent’s Bioanalyzer. The results showed that BioMérieux (EM) eluates contained intact rRNA, while Norgen and Qiagen eluates revealed possible rRNA degradation or lower recovery of the larger rRNA molecules (Supplementary Fig. S1). A control sample of bacterial and human RNA showed that the rRNA peaks in the electropherograms from the patient stool samples largely reflected procaryotic RNA, making them unsuitable for qualitative inspection of eucaryotic RNA (Supplementary Fig. S1).

### Comparison of RT-PCR methods

**Fig. 4:**
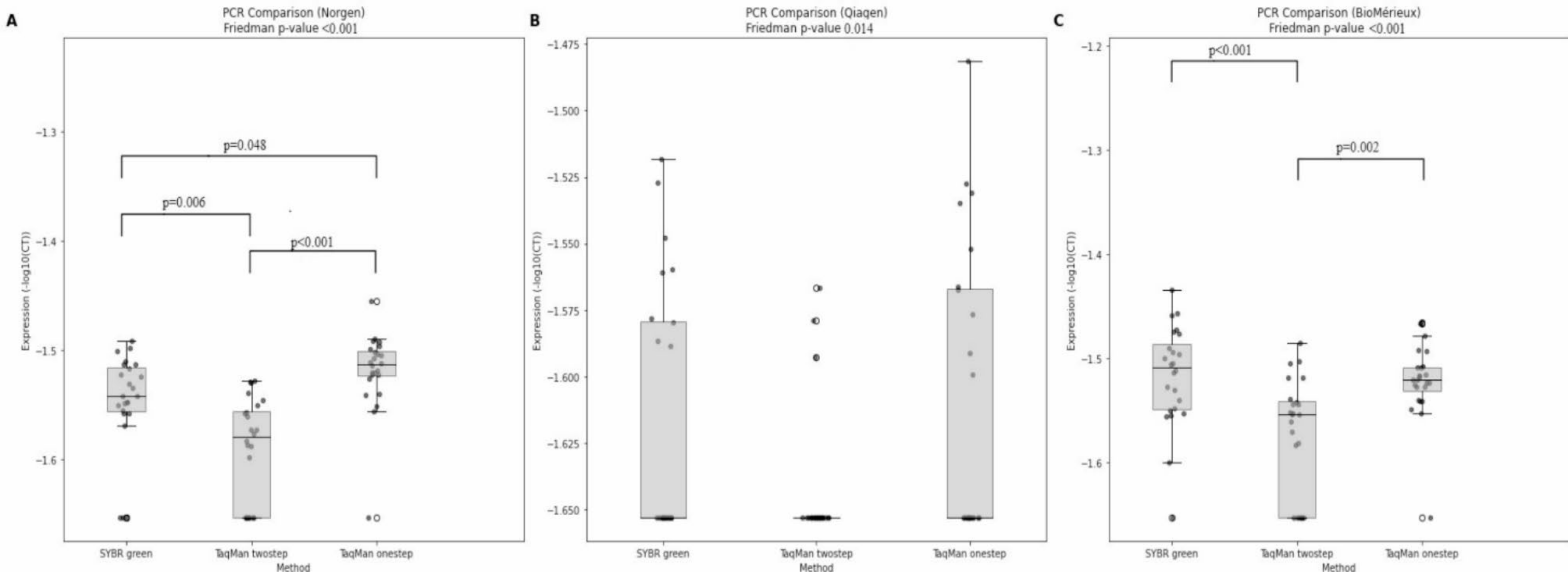
*GAPDH* transcript detection comparison using three RNA extraction and three PCR methods. (**A**) Illustrates *GAPDH* transcript detection levels from RNA extracted using the Norgen method and analyzed with SYBR green, TaqMan two-step, and TaqMan one-step PCR methods. Significant differences in detection levels were noted between SYBR green and TaqMan two-step (*p* = 0.048), SYBR green and TaqMan one-step (*p* < 0.001), and TaqMan two-step and TaqMan one-step (*p* = 0.006), with the highest detection observed with the TaqMan one-step method. (**B**) Illustrates the levels of *GAPDH* transcript detection obtained from RNA extracted using the Qiagen method. There was no difference in the levels of transcripts determined by the SYBR green and TaqMan one-step PCR methods. The TaqMan two-step assay exhibited low detection levels. (**C**) Illustrates *GAPDH* transcript detection levels from RNA extracted using the BioMérieux method. Significant differences were observed between SYBR green and TaqMan twostep (*p* < 0.001), SYBR green and TaqMan one-step (*p* = 0.002), and TaqMan twostep and TaqMan one-step methods (*p* < 0.001).

To determine the most sensitive RT-PCR protocol for the detection of *GAPDH* transcripts in stool, RNA prepared by all three extraction protocols were tested. We compared the performance of three RT-PCR protocols: SYBR green two-step, TaqMan two-step, and TaqMan one-step (Fig. 4; Table 4). Friedman test showed a notable disparity in gene transcript levels determined by the three PCR methodologies across the three kits (Norgen: *p* < 0.001, Qiagen: *p* = 0.014, BioMerieux: *p* < 0.001). The TaqMan one-step approach generally resulted in lower Ct-values in comparison to the SYBR green and TaqMan two-step procedures. Using RNA from the Norgen extraction method, TaqMan one-step outperformed both SYBR green and TaqMan two-step, demonstrating significantly higher sensitivity (p-values < 0.05) (Table 4).

### Quality controls

None of the negative controls were amplified in any of the assays. The specificity of our PCR reactions was confirmed by agarose gel electrophoresis, which showed distinct bands of the estimated sizes, and by melting curve analysis, which showed single peaks. The RNA extraction control cel-miR-39 “spike-in” showed that the Qiagen and Norgen techniques reliably recovered miRNA with high efficiency (Supplementary Table S1), However, the EM method did not extract small-sized RNA as effectively as the other two protocols, which is a well-documented phenomenon in silica-coated bead-based extraction^43^. The 200-base-pair IC-oligo cDNA synthesis/PCR control was consistently detected in samples extracted by EM and Norgen but showed large variation in samples extracted by Qiagen, from which five samples yielded no signal, while the remaining samples showed high signals. (Supplementary Table S2).

**Table 4:** Comparison of extraction methods and PCR methods (Dunn’s test).

Comparison	Group A	Group B	Median (A)	Median (B)	*P*-value
Extraction RNA Qubit (Conc.ng/µl)	BioMérieux	Norgen	35.9	23.3	1.000
	BioMérieux	Qiagen	35.9	7.7	0.023
	Norgen	Qiagen	23.3	7.7	0.003
Extraction RNA nanodrop (Conc.ng/µl)	BioMérieux	Norgen	17.7	62.6	< 0.001
	BioMérieux	Qiagen	17.7	22.7	1.000
	Norgen	Qiagen	62.6	22.7	0.006
Extraction nanodrop RNA purity (A260/A280)	BioMérieux	Norgen	1.3	2.0	< 0.001
	BioMérieux	Qiagen	1.3	1.8	< 0.001
	Norgen	Qiagen	2.0	1.8	0.154
Extraction nanodrop RNA purity (A260/A230)	BioMérieux	Norgen	0.4	1.8	< 0.001
	BioMérieux	Qiagen	0.4	0.3	1.000
	Norgen	Qiagen	1.8	0.3	< 0.001
*GAPDH* PCR Norgen (CW*)	SYBR green	TaqMan one-step	1.54	1.51	0.048
	SYBR green	TaqMan two-step	1.54	1.58	0.007
	TaqMan one-step	TaqMan two-step	1.51	1.58	< 0.001
*GAPDH* PCR Qiagen (CW*)	SYBR green	TaqMan one-step	1.65	1.65	1.000
	SYBR green	TaqMan two-step	1.65	1.65	0.144
	TaqMan one-step	TaqMan two-step	1.65	1.65	0.057
*GAPDH* PCR BioMérieux (CW*)	SYBR green	TaqMan one-step	1.51	1.52	1.000
	SYBR green	TaqMan two-step	1.51	1.55	0.001
	TaqMan one-step	TaqMan two-step	1.52	1.55	0.002

* Efficiency-corrected Ct values (CW).

### mRNA transcript levels of CRC-associated genes in cancer and polyp patients and controls

**Fig. 5:**
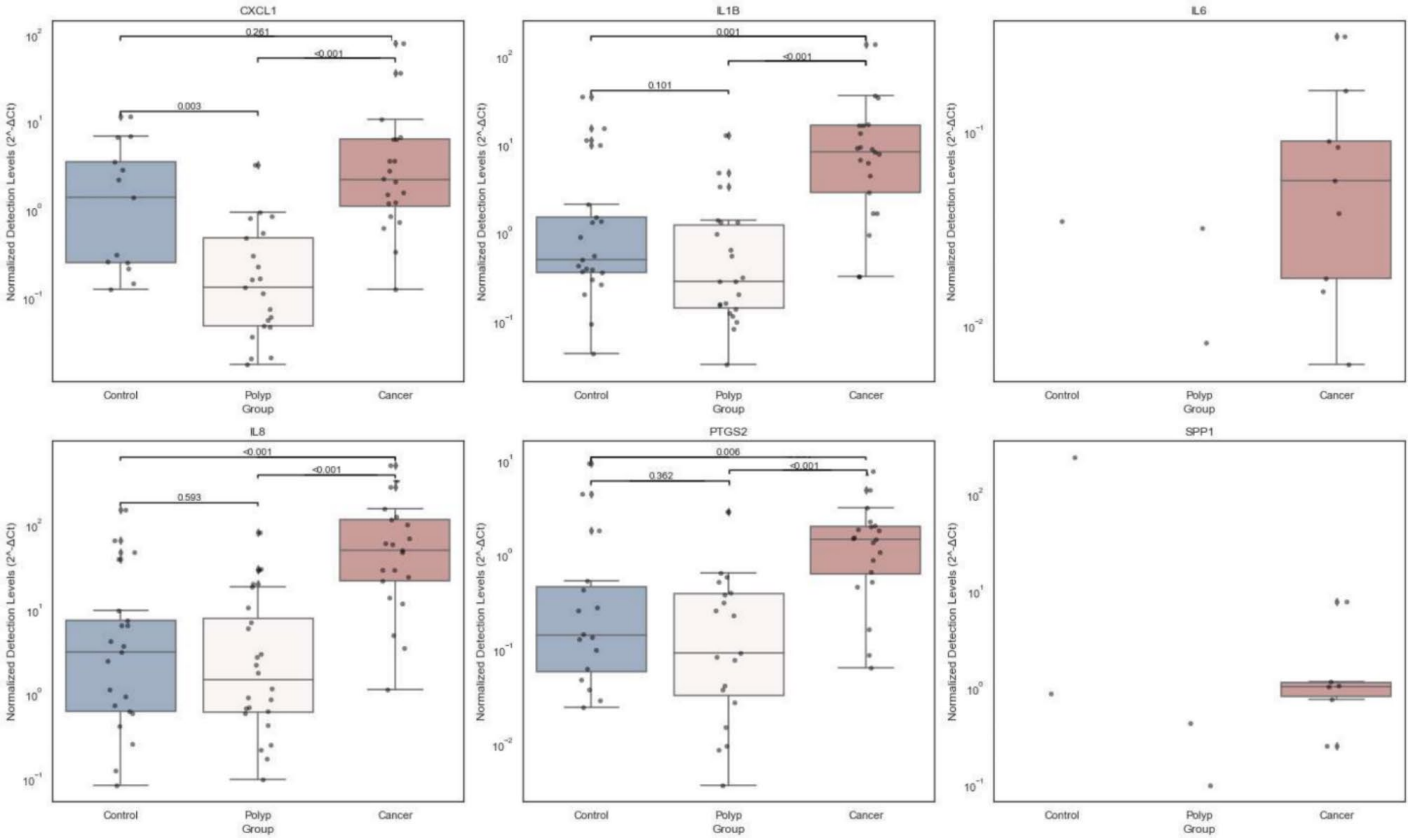
Comparison of mRNA levels of inflammatory genes in control, polyp, and cancer groups. This figure presents box plots of normalized transcript levels 2^−ΔCt^ for six inflammatory markers (*CXCL1*, *IL1B*, *IL6*, *IL8*, *PTGS2*, and *SPP1*) across three groups: control(blue), polyp (white), and cancer(red). The statistical significance of differences between groups is indicated by p-values.

The combination of Norgen RNA extraction and TaqMan one-step PCR was evaluated as the optimal protocol for measuring *GAPDH* transcript levels in stool samples. We therefore conducted one-step RT-PCR analysis of fecal samples extracted with the Norgen kit from all 68 patients in the test cohort—control, polyp, and cancer (Table 1). The objective was to evaluate the protocol using immune genes associated with colorectal cancer in stool samples^17^. Specifically, we examined the expression levels of the genes *CXCL1*, *IL8*, *IL1B*, *IL6*, *PTGS2*, and *SPP1*, assessing their utility for non-invasive CRC detection (Fig. 5).

The results showed that transcripts from all investigated genes were identified in stool samples from the majority of all patient groups. This confirms the effectiveness of the extraction and RT-PCR protocols employed for the detection of multiple mRNA in stool samples. *CXCL1*, *IL8*, *IL1B*, and *PTGS2* were detected in all three patient groups, presenting significant differences in transcript levels between the groups. The Kruskal-Wallis rank sum test was used to assess the variations in the groups and within each group. The results of the Kruskal-Wallis test revealed a statistically significant difference between the groups for all genes in both tumors and polyps (*p* < 0.001), with the exception of *IL6* and *SPP1* (Supplementary Table S3). Finally, we conducted group comparisons using the Mann-Whitney U test and corrected the P values using the Bonferroni correction. *IL6* and *SPP1* transcripts were only detected in nine and eight cancer samples, respectively, and were nearly absent in polyp patients and controls, suggesting that transcript levels were generally lower and more cancer-specific, or that these RT-PCR assays were less efficient. *IL1B*, *IL8* and *PTGS2* were significantly higher in stool from cancer patients compared to the polyp and control group (*p* < 0.05). There was no significant difference between polyps and controls for these genes. *CXCL1* showed non-significant levels of detection in the cancer group compared to the control group. However, *CXCL1* was significantly higher in stool from cancer patients compared to the polyp group (*p* < 0.001) (Fig. 5).

## Discussion

Due to the immune system’s involvement in CRC progression, the mRNA transcripts from immune genes may hold potential for non-invasive detection of the disease in its early stages^17,44,45^. Emerging evidence supports the use of mRNA-based approaches for non-invasive detection of CRC lesions, with mRNA in stool now considered a suitable biomarker^27^. However, there is no consensus on mRNA detection methods in feces, leading to varying research protocols^24,27,31^.

The primary objective of this study was to evaluate the impact of different extraction methods and RT-PCR protocols for mRNA detection. We assessed three commercial RNA extraction kits and protocols that represented different methods for RNA extraction: one paramagnetic bead-based method with the EasyMAG instrument from BioMérieux and two manual spin column-based methods with different RNA-binding technologies: a silica gel in the Qiagen spin column and a resin separation matrix in the Norgen spin column. The highest RNA yields and purities were obtained with the Norgen kit. This kit is specifically developed for stool samples and does not contain any organic solvents or guanidine-based buffers (Table 2), which may explain the high purity of RNA. This kit has also shown good results in other studies, e.g., a recent multicenter study with promising results for a fecal miRNA signature for CRC patients using RNA extracted with the Norgen kit^46^. On the negative side, the Norgen kit required manual sample processing, and the resin columns occasionally occluded, a stage in the process that warrants enhancement by the manufacturer. In this regard, the stool protocol on the EasyMAG instrument had a faster turn-around time and a lower workload for the laboratory technician (Table 2). However, no specific RNA protocol was available on this instrument, and a manual DNAse treatment subsequent to the extraction was therefore required. Consequently, the time saved with Biomérieux (EM) was limited. Furthermore, the eluates from EM resulted in low purities, represented by low A260/280 and A260/230 ratios. Low A260/230 ratios were also observed from RNA extracted with the Qiagen kit (Fig. 3), suggesting chemical contamination that could potentially affect downstream applications^47^. RNA from the Qiagen kit revealed that neither *GAPDH* transcripts nor the IC-oligo were detected consistently between the samples, suggesting incomplete removal of inhibitors from stool or incompatibility with the RT- or PCR-reagents^47^. All factors considered, the Norgen kit was evaluated as the best-performing extraction kit in the present study.

In addition to testing mRNA extraction methods, we also evaluated different RT-PCR protocols using *GAPDH* as a target. The three protocols represented two different detection chemistries: double-stranded DNA-intercalating dyes (SYBR green) and TaqMan hydrolysis probes. In addition, our experimental design incorporated a two-step RT-PCR, which involves separate reverse transcription and PCR steps, as well as a one-step RT-PCR protocol, wherein both reactions are conducted within the same tube and program. The one-step RT-PCR protocol utilizing Superscript III reagents and TaqMan probes yielded the most optimal *GAPDH* detection. This protocol uses target-specific primers only, also for the reverse transcription. A concern with one-step RT-PCR is that the total amount of RNA may affect the efficacy of the reverse transcription and also that it is less flexible for the storage and archiving of material^48^. However, others have shown that the stability of mRNA and cDNA is considered similar^49^. Furthermore, it has been shown that one-step protocols are more sensitive for less expressed genes^49^. Our data are in line with these observations. A limitation of the present study is that different primer targets were used for the SYBR green protocol and the probe-based assay, and that differences between the two may be due to different primer efficiencies, limiting the protocol comparison. The performance of the SYBR green protocol was comparable to the one-step TaqMan protocol and may therefore successfully be used for mRNA detection studies in stool samples. The one-step protocol was, however, easier to perform and required less hands-on time, and was therefore the preferred protocol in our hands.

In summary, our results showed that the combination of the Norgen extraction method with the TaqMan one-step PCR method was the most effective, providing high RNA yields, good purity, and sensitive and consistent mRNA detection. To facilitate the use of the manual Norgen extraction methods in large-scale studies, the protocol could be transferred to a pipetting robot for automated execution of the different steps.

In the second part of the study, the chosen protocol was tested on another cohort of samples consisting of 22 stool samples from cancer patients, 24 samples from patients with adenomatous polyps, and 22 controls without any neoplastic findings during colonoscopy. Detection levels of six inflammatory markers (*CXCL1*, *IL1B*, *IL6*, *IL8*, *PTGS2*, and *SPP1*) previously associated with CRC were compared between the groups. Our results demonstrated significantly higher detection levels in colorectal cancer patients compared to the other two groups for all genes except *CXCL1*, which did not show any statistical difference between cancer patients and controls. Our results therefore confirm the successful use of the applied protocol and strongly support the use of stool as a sample material for the detection of mRNA.

*IL1B* showed marked increases in the cancer group, highlighting their association with tumorigenesis and inflammation, consistent with previous findings^50^. Levels of *IL8* and *PTGS2* transcripts were also significantly increased in the cancer group, reinforcing their association with cancer-related inflammation^51,52^. *SPP1* and *IL6* followed a similar trend, with only elevated detection levels in cancer, suggesting their involvement in colorectal cancer^53,54^.

Interestingly, the control samples also showed elevated transcript levels of some genes, which could be attributed to various factors. The patients undergoing colonoscopy in the present study were selected for the procedure due to various gastrointestinal symptoms or conditions, such as gastrointestinal bleeding, weight loss, and changes in bowel habits. These underlying conditions themselves could also affect gene expression.

This study detected highly elevated levels of transcripts of the targeted immune genes in the fecal samples. The reason for this could be that the samples were preserved in RNAlater before mRNA extraction, which may have improved the sensitivity of mRNA detection. RNAlater efficiently preserves the integrity of RNA in biological samples, effectively preventing its degradation during the processes of collection, storage, and transport. It is necessary to preserve stool samples, as they contain RNases and other enzymes that degrade RNA. Previous studies have shown that RNAlater is effective in preserving RNA stability in different types of biological samples, even when stored at room temperature. This finding reinforces the reliability of the method^55–57^.

The number of samples investigated in the present study was only 22–24 in each group. We therefore cannot draw any conclusions on whether these genes may be used as biomarkers for CRC detection. For this purpose, a similar study must be performed on a larger cohort of patient samples. Several of the immune genes tested in this study are also known to be increased in other intestinal diseases, in particular inflammatory bowel disease (IBD); therefore, for the purpose of biomarkers, these genes may be used in combination with a larger panel, possibly consisting of miRNA, bacterial genes, and mutation analysis.

## Conclusion

By highlighting the strengths and limitations of different RNA extraction methods and PCR techniques, we offer practical recommendations for optimizing RNA quality and gene transcript detection in stool. Our results confirm the successful use of stool as a sample material for detection of CRC associated mRNA. Future studies should focus on validating these findings in larger cohorts and exploring the potential of using the mRNA of immune genes, related to CRC, as non-invasive biomarkers in stool samples.

## Electronic supplementary material

Below is the link to the electronic supplementary material.

Supplementary Material 1.

## Data Availability

The original contributions presented in the study are included in the article/Supplementary Material. Further inquiries can be directed to the corresponding author.
